# Effect of pedicle-screw rod fixation on oblique lumbar interbody fusion in patients with osteoporosis: a retrospective cohort study

**DOI:** 10.1186/s13018-021-02570-8

**Published:** 2021-07-03

**Authors:** Kaiwen Cai, Kefeng Luo, Jinjin Zhu, Kai Zhang, Shengkai Yu, Yi Ye, Guoqiang Jiang

**Affiliations:** 1 Department of Orthopaedic, The Affiliated Hospital of Medical School of Ningbo University, No. 247, Renmin Road, Jiangbei District, Ningbo, Zhejiang, People’s Republic of China; 2 Institute of Orthopaedics, Ningbo University, Ningbo, People’s Republic of China; 3 Department of Orthopaedic, Sir Run Run Shaw Hospital, School of Medicine, Zhejiang University, Hangzhou, People’s Republic of China; 4 The Medical School of Ningbo University, Ningbo, People’s Republic of China

## Abstract

**Study design:**

A retrospective cohort study.

**Objective:**

To investigate the radiological and clinical outcomes of patients with or without pedicle-screw rod fixation (PSRF) in OLIF surgery.

**Methods:**

Between June 2017 and December 2019, 66 consecutive patients who underwent OLIF surgery at two centers were divided into stand-alone and combined groups according to whether or not PSRF was used. Imaging and clinical data were collected preoperatively, postoperatively, 3 and 6 months postoperatively, and at the last follow-up. Related coefficient and multiple linear regression analysis was used to detect the influencing factors of cage subsidence (CS).

**Results:**

There was a lower baseline BMD in the combined group (p = 0.005). The combined group showed superior VAS score at 3 months postoperatively, although there was no difference in long-term VAS and ODI scores between the two groups. The foraminal height (FH) of the two groups was comparable at preoperatively, postoperatively, and 3 months postoperatively, but the combined group showed better maintenance of FH at 6 months postoperatively (p = 0.049) and last follow-up (p = 0.019). The total CS (tCS) of the combined group was lower than that of the stand-alone group during the whole follow-up period (all p ≤ 0.001). Multiple linear regression suggested that lower BMD was the risk factor for main CS, and PSRF could significantly reduce the BMD threshold for severe CS (−4.77 vs −1.38).

**Conclusions:**

OLIF combined with PSRF can effectively avoid foraminal height loss and prevent severe CS, which may be more suitable for patients with osteoporosis or osteopenia and improve clinical outcomes.

## Background

Oblique lumbar interbody fusion (OLIF) has become a widely used indirect decompression and interbody fusion technique in the last decade. The rudiment of the OLIF operation method was described by Dr. Mayer [1] in 1997, who established a working channel between the left psoas major muscle and the abdominal vascular sheath and completed intervertebral fusion via this channel. Due to its minimally invasive advantages and allowing the implantation of a larger cage [2, 3], it has become one of the main surgical methods for the treatment of degenerative lumbar instability.

Because it is believed that the large size of the cage can provide adequate stability, many scholars have used the stand-alone OLIF method to stabilize the intervertebral space and achieved good clinical results [4–6]. However, the stability of stand-alone OLIF has attracted attention and been researched recently. Fang G et al. reported that the segment stability of the stand-alone OLIF was worse than that of the OLIF combined with bilateral pedicle screw fixation method; endplate stress was more close to the yield stress of the lamellar bone that means the patients who underwent stand-alone OLIF might face a greater risk of cage subsidence (CS) [7]. Remarkable CS is considered to be significantly related to worse clinical outcomes [8]. What’s worse, existing studies have shown a non-negligible incidence of CS during OLIF [8–11].

Based on experience, risk factors for CS include endplate injury, osteoporosis, grade II spondylolisthesis or above, isthmic spondylolisthesis, and multilevel fusion [3]. To avoid this risk, additional fixation was introduced for the OLIF procedure, resulting in combined OLIF. Among all additional implantation, pedicle-screw rod fixation (PSRF) is the most commonly used, not only it disperses the stress transfer and protects the endplate but it also increases the operation time, medical cost, intraoperative bleeding amount, and invasion of paraspinal tissue. At present, the evidence on the risks and benefits of stand-alone/PSRF is mixed, and it is still not clear when additional PSRF is necessary in OLIF surgery.

The purpose of this study was to clarify the effect of PSRF on preventing CS in OLIF surgery and to further clarify the indications of its use.

## Methods and materials

This study was approved by the clinical ethics committee of The Affiliated Hospital of Medical School of Ningbo University (KY20200906). Due to the retrospective nature of this study, the written informed consent of patients was waived.

### Patient selection

From June 2017 to December 2019, 94 consecutive patients underwent OLIF with or without PSRF surgery in two centers; the medical records of them were reviewed. The inclusion criteria were as follows: (1) Patients who underwent OLIF surgery with or without PSRF, (2) conservative treatment was ineffective for 3 months, and (3) pain symptoms are mainly low back pain, radiculotic symptoms are not or mild. The exclusion criteria were as follows: (1) Patients combined with any direct decompression, (2) patients with incomplete radiological data, (3) patients who were lost or had incomplete follow-up records, (4) patients received a change in the implantation method for revision surgery, and (5) multilevel surgery.

As a result, sixty-six patients were enrolled. The average follow-up period was 22.6 ± 6.6 months (range 13-34 months). Among the 66 patients included, 41 patients who received stand-alone OLIF were set as the stand-alone group, and 25 patients who received OLIF combined with bilateral PSRF were set as the combined group. Among the 28 excluded patients, 17 cases were lost, 8 cases had incomplete radiography data, and 3 were withdrawn from the cohort because of secondary pedicle screw fixation after primary stand-alone surgery due to unbearable low back pain. The demographic baseline characteristics of the patients including age, gender, body mass index (BMI), and bone mineral density (BMD) are described in Table 1. There was no significant difference in age, sex, or BMI between the two groups, but the BMD in the combined group was significantly lower (−1.9 ± 1.3 vs −0.9 ± 1.4, p = 0.005).

**Table 1:** Baseline characteristics of included patients

Baseline data	Combined (n = 25)	Stand-alone (n = 41)	P value
Age (year)	62.16 ± 8.65	59.46 ± 8.46	0.217
Gender (M/F)	12/13	22/19	0.800
BMI (kg/m^2^)	24.32 ± 1.59	24.33 ± 2.40	0.984
BMD (T value)	−1.9 ± 1.3	−0.9 ± 1.4	0.005
Surgical segment			
L_2/3_	0	2	0.532
L_3/4_	5	11	
L_4/5_	20	28	
L_5_/S_1_	0	0	

*BMI* body mass index, *BMD* bone mineral density

### Surgical management

All surgeries were performed by senior spine surgeons with more than 20 years of experience and their teams. Generally, after general anesthesia, the patients were placed in the right lateral position, and a standard OLIF procedure was performed, as described in the previous literature [4–6]. In patients who underwent combined OLIF, additional percutaneous PSRF was performed as described in previous studies [12, 13]. No direct decompression of the spinal canal was performed.

After the operation, all patients received nonsteroidal drugs, muscle relaxants, and neurotrophic drugs. Lumbar and leg muscle training was conducted under the guidance of physiotherapists. In addition, patients with BMD values between −1.5 and −2.5 received calcium and calcitriol, while patients with BMD values lower than −2.5 received bisphosphonates in addition to the above drugs.

### Clinical outcome data collection

The visual analog scale (VAS) [14] was used to evaluate low-back pain, which was recorded preoperatively, postoperatively, 3 and 6 months postoperatively, and at the last follow-up. Oswestry disability index (ODI) [15] is also measured and recorded at these time points. Surgical-related complications were documented.

### Radiography data measurement

**Fig. 1:**
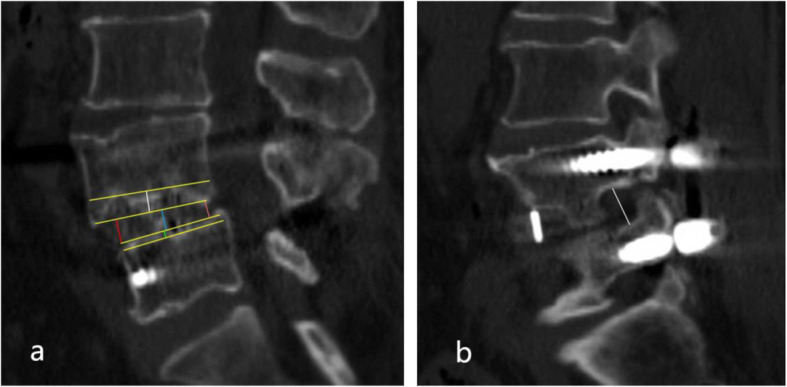
Measurement of radiological parameters: (**a**) the boundary of the endplate and the cage is delineated by yellow line. The tCS is defined as the sum of the cephalic CS (white line) and caudal CS (green line), and mCS is the larger one in them. DH is defined as the mean of the anterior DH (red line), middle DH (blue line), and posterior DH (pink line). (**b**) FH is measured by the perpendicular from the lower edge of the cephalic vertebra pedicle to the upper edge of the caudal vertebra pedicle. FH is the mean value of bilateral FH

The radiography measurements and interbody fusion evaluations were evaluated by sagittal lumbar computed tomography. We measured the disk height (DH) of each patient’s operative segment. The DH was defined as the mean value of the anterior, middle, and posterior heights of the intervertebral space (Fig. 1). The ΔDH was defined as the postoperative DH minus the preoperative DH, which represents the increment in the height of the intervertebral space. The foraminal height (FH) was defined as the mean of bilateral foraminal height, measured perpendicular from the lower edge of cephalic pedicle to the upper edge of caudal pedicle, it indicate the effect of foraminal decompression. The total cage subsidence (tCS) was calculated as the sum of cephalic and caudal subsidence (Fig. 1). Since the tCS is composed of two variable whose length is opposite to that of the cephalic and caudal, and the correlation between CS and clinical outcomes depends mainly on the severity of single CS rather than the sum. Thus, the one with the larger value is considered as the main cage subsidence (mCS). Severe CS is considered to occur when mCS > 4 mm [16]. The evaluation of fusion rate at 6 months postoperatively and at the last follow-up was performed using the Bridwell interbody fusion grading system [17]. If grade I or grade II was reached, the occurrence of interbody fusion was considered. All imaging data were independently and blindly evaluated by two senior radiologists. Controversial measurement results were alleviated by consensus.

### Statistical analysis

SPSS 23.0 software (IBM Co., Armonk, NY, USA) was used to analyze the data. One-sample K-S test was used to check the normality of the measurement data, and the mean ± SD was used if normality was met. If the data did not obey a normal distribution, we described the variables as M [Q_1_, Q_3_], and the Wilcoxon Mann-Whitney test was used. Fisher’s exact probability method was used to compare the enumeration data between groups. For correlation analysis, related coefficient and multiple linear regression analyses were used. The test standard was set as α = 0.05.

## Results

### Clinical outcomes

**Fig. 2:**
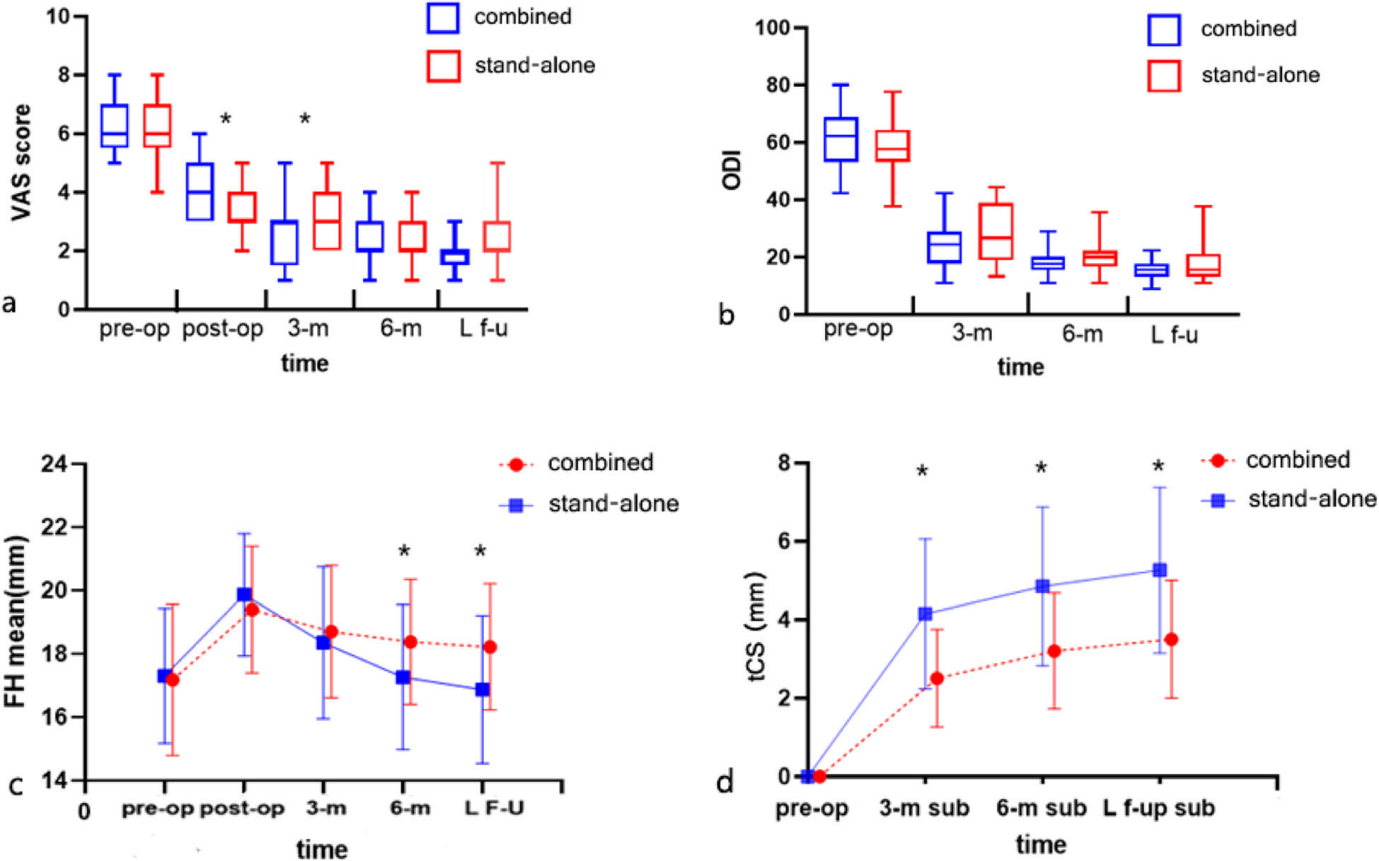
The clinical and radiological results of the two groups. (**a**) VAS comparison between groups. (**b**) ODI comparison between groups. (**c**) FH at each time point of the two groups. (**d**) tCS at each time point of the two groups. * The difference was statistically significant (p < 0.05)

Before surgery, there was no significant difference in the VAS (6 [5.5, 7] vs 6 [5.5, 7], p = 0.826) or ODI (62.22 [53.33, 68.89] vs 57.78 [53.33, 64.44], p = 0.551) scores between the combined group and stand-alone group. The postoperative VAS and ODI scores decreased significantly in each group and were all maintained well in whole follow-up period. Immediate postoperatively, the VAS score of the stand-alone group was slightly better than that of the combined group (3 [3, 4] vs 4 [3, 5], p = 0.012). However, the VAS score in the combined group was better than that in the stand-alone group 3 months after the operation (3 [1.5, 3] vs 3 [2, 4], p= 0.038). The difference disappeared at 6 months postoperatively and at the last follow-up. There was no significant difference in ODI scores between the groups at any of the time points (Table 2, Fig. 2).

**Table 2:** Clinical outcomes of the two groups

Groups	VAS score					ODI (%)			
	Pre-op	Post-op	3 m	6 m	Last f-u	Pre-op	3 m	6 m	Last f-u
Combined	6 [5.5, 7]	4 [3, 5]	3 [1.5, 3]	2 [2, 3]	2 [1.5, 2]	62.22 [53.33, 68.89]	24.44 [17.78, 28.89]	17.78 [15.56, 20.00]	15.56 [13.33, 17.78]
Stand-alone	6 [5.5, 7]	3 [3, 4]	3 [2, 4]	2 [2, 3]	2 [2, 3]	57.78 [53.33, 64.44]	26.67 [18.89, 38.89]	20.00 [16.67, 22.22]	15.56 [13.33, 21.11]
Z value	−0.229	−2.528	−2.075	−1.657	−1.823	−0.603	−1.348	−1.134	−0.960
P value	0.826	0.012	0.038	0.100	0.067	0.551	0.180	0.260	0.341

*VAS* visual analog scale, *ODI* Oswestry disability index

### Radiography outcomes

For all patients, the DH increased by 3.11 [2.12, 4.45] mm after the operation, but there was no significant difference in the ΔDH between the two groups (3.24 [2.03, 4.55] vs 3.08 [2.15, 4.33], p = 0.848). The FH values preoperatively and 3 months postoperatively were comparable between the two groups. There was a significant difference of FH between the two groups 6 months after the operation (18.37 ± 1.98 vs 17.26 ± 2.29, p = 0.049), and the difference was more significant at the last follow-up (18.22 ± 2.00 vs 16.87 ± 2.34, p = 0.019). The combined group had significantly lower tCS than the stand-alone group at all time points after the operation (all p ≤ 0.001). The occurrence of severe CS of combined group was lower than that of stand-alone group, but the difference was not significant (16% vs 41.5%, p =0.055). There was no significant difference in the fusion rate between the two groups 6 months after the operation or at the last follow-up (92% vs 90.2%, 100% vs 95.1%, respectively) (Table 3, Fig. 2).

**Table 3:** Radiological results the two groups

Groups	ΔDH (mm)	FH (mm)					tCS (mm)			Severe CS (> 4 mm)	Fusion rate	
		Pre-op	Post-op	3 m	6 m	Last f-u	3 m	6 m	Last f-u		6 m	Last f-u
Combined	3.24 [2.03, 4.55]	17.18 ± 2.39	19.40 ± 2.00	18.71 ± 2.10	18.37 ± 1.98	18.22 ± 2.00	2.50 ± 1.24	3.20 ± 1.48	3.50 ± 1.50	4/25 (16%)	23/25 (92%)	25/25 (100%)
Stand-alone	3.08 [2.15, 4.33]	17.31 ± 2.14	19.87 ± 1.93	18.36 ± 2.41	17.26 ± 2.29	16.87 ± 2.34	4.15 ± 1.91	4.85 ± 2.03	5.26 ± 2.11	17/41 (41.5%)	37/41 (90.2%)	39/41 (95.1%)
Statistical value	Z= -0.192	t= −0.226	t= −0.955	t= 0.599	t= 2.004	t= 2.397	t= −3.837	t= −3.522	t= −3.641	**\**	**\**	**\**
P value	0.848	0.822	0.343	0.551	0.049	0.019	< 0.001	0.001	0.001	0.055	1.000	0.522

*DH* diskal height, *FH* foraminal height, *tCS* total cage subsidence

### Correlation analysis

In the combined group, BMD was mildly negatively correlated (r = −0.602, p = 0.001) with mCS, and BMI was a moderate-intensity positive correlation for mCS (r = 0.400, p = 0.047), while ΔDH (r =−0.059, p = 0.781) were not significantly correlated with mCS. In contrast, in the stand-alone group, the negative correlation between BMD and mCS was significantly enhanced (r = −0.797, p < 0.001), and a correlation between mCS and BMI (r = 0.207, p = 0.195) or ΔDH (r = 0.271, p = 0.086) was not found.

On the other hand, there was only a low to moderate positive correlation between mCS and VAS (r = 0.427, p = 0.033) or ODI (r = 0.594, p = 0.002) scores in the combined group. However, in the stand-alone group, this positive correlation was much stronger with VAS (r = 0.685, p < 0.001) and DOI (r = 0.616, p < 0.001).

Multiple linear regression showed that only BMD was a risk factor for mCS in the regression model in the combined group (β = −0.535, p = 0.005) and stand-alone group (β = −0.756, p < 0.001) (Table 4). Therefore, BMI and ΔDH were excluded from the multiple linear regression model, and a simple linear regression model of BMD and mCS was used (Table 5). According to the linear regression model, when severe CS was defined as mCS > 4 mm, the BMD threshold in the stand-alone group was −1.38, and the use of PSRF expanded the BMD threshold to −4.77 in the combined group.

**Table 4:** Multiple linear regression results

Groups	Variables	SE	β	95%CI	P value
Combined	Constant	3.003		(−9.539, 2.950)	0.285
	BMD	0.156	−0.535	(−0.812, −0.164)	0.005
	BMI	0.125	0.268	(−0.063, 0.457)	0.130
	ΔDH	0.085	−0.002	(−0.177, 0.176)	0.992
Stand-alone	Constant	1.528		(−3.688,2.505)	0.701
	BMD	0.102	−0.756	(−1.031, −0.618)	< 0.001
	BMI	0.060	0.185	(−0.002, 0.240)	0.054
	ΔDH	0.084	0.177	(−0.013, 0.329)	0.069

*Dependent variable* mCS, *SE* standard error, *95% CI* 95% confidence interval

**Table 5:** Simple linear regression results

Groups	Variables	SE	β	95%CI	P value
Combined	Constant	0.342		(0.675, 2.090)	0.001
	BMD	0.152	−0.602	(−0.862, −0.234)	0.001
Stand-alone	Constant	0.174		(2.451, 3.153)	< 0.001
	BMD	0.106	−0.797	(−1.083, −0.656)	< 0.001

*Dependent variable* mCS, *SE* standard error, *95% CI* 95% confidence interval

### Complications

No cases of major artery injury or spinal nerve injury occurred. One case of segmental artery injury occurred in the stand-alone group, and lumbar myasthenia was relatively more common, with 4 cases occurring in each group. There were 3 cases of sympathetic nerve injury in the combined group and 2 cases in the stand-alone group. There was 1 case of ureteral injury in the stand-alone group. No cage retropulsion or other complications were observed in either group. Statistical analysis was abandoned due to the small number of complications.

## Discussion

As a kind of anterolateral fusion, the decompression effect of OLIF is indirect, which also leads to the limitation of surgical indications. Generally, the surgical indications of OLIF are lumbar spondylolisthesis below grade II, lumbar disk herniation combined with segmental instability, nerve root canal stenosis caused by loss of disk height, degenerative scoliosis/kyphosis, and degenerative disk disease [18]. Therefore, patients now have a more minimal invasive choice of fusion. To pursue the minimally invasive advantage of OLIF, stand-alone OLIF is widely used, as it has a shorter operation time and less intraoperative bleeding than other operations and does not invade the posterior structure [19].

However, stand-alone OLIF method is controversial due to its potential stability defects. Fang G et al. [7] and Guo Hz et al. [20] found that the stability of stand-alone OLIF was significantly worse than that of combined OLIF through finite element analysis. In contrast, St Clair s et al. proved that the stability, overall stiffness, and failure load of stand-alone OLIF were similar to those of pedicle screw rod fixation systems through biomechanical testing of the human spine in vitro [21]. In clinical studies, the comparison results of stand-alone OLIF and combined OLIF have also been inconsistent. In some studies, the short-term clinical results of stand-alone OLIF were better [9, 22], but in other studies, the complication rate of stand-alone OLIF was significantly higher than that of combined OLIF [10, 11]. Therefore, the evidence on the risks and benefits of stand-alone/PSRF method remains mixed.

In addition, the indications of PSRF are ambiguous, some studies have only empirically suggested that patients with a BMD < −1 should undergo pedicle screw fixation to prevent subsidence [3]. Other authors believe that additional fixation may be required for patients with a BMD < −2.5 or who with BMI ≥ 30 kg/m^2^ [23]. At present, there is no consensus on when additional implantation is necessary in OLIF surgery.

Biomechanically, stiff support will transfer more gravity load between vertebrae than soft disk tissue before bony fusion achieved. Although the cages used in OLIF are larger than traditional cages, the stress shielding effect is still almost unavoidable in stand-alone fusion [24]. Excessive stress leads to local collapse of the endplate followed by repeated trabecular microfractures under the endplate in daily activities and causes intractable pain [25]. This process gradually stops until the occurrence of curvature coincidence, sub-endplate osteosclerosis, and intervertebral fusion.

In our study, we found a strong negative correlation between BMD and CS, indicating that osteoporosis patients are more prone to the occurrence of CS. The correlation analysis also confirmed a correlation between a larger mCS and worse VAS and ODI scores. PSRF showed an obvious anti-subsidence effect, and can weaken the positive correlation between mCS and VAS/ODI. Previously, Oh kW et al. [26] proved that there was no correlation between CS and clinical results after PLIF surgery. Similarly, we also found that PSRF could prevent the low back pain and disability caused by CS, for the correlation between VAS/ODI and CS was significantly weakened in the combined group. These results demonstrated the effect of interbody stabilization on pain relief.

The ephemeral inferiority in VAS scores immediate postoperatively in the combined group may be caused by additional posterior surgical trauma. From 6 months to the last follow-up, because interbody fusion was achieved in the majority of patients in both groups, the unstable factors of the intervertebral space were eliminated, induce the difference in pain improvement between the groups disappeared. In terms of functionality improvements, although the ODI scores in the combined group were always slightly better than those in the stand-alone group, there was no significant difference between the two groups.

In this study, although the difference of the incidence of severe CS between the two groups was not significant (p = 0.055), we noted that this was due to the significant difference in baseline BMD value between the two groups caused by the non-randomized grouping. If BMD value was similar between the groups, this difference would be further amplified. The correlation analysis showed that osteoporosis was a risk factor for mCS. According to the linear regression models obtained in this study, patients who underwent the stand-alone method were at risk of severe CS when their BMD < −1.38. When PSRF was adopted, only patients with a BMD < −4.77 were at equal risk of severe CS.

Interestingly, we found that the BMI did not show a correlation with mCS in any group, which may be because, on the one hand, greater BMI will increase vertebral load as a risk factor, and on the other hand, greater BMI is a protective factor for osteoporosis in postmenopausal women [27, 28]. Although the P value of BMI in both groups was greater than 0.05, we found that the P value of the stand-alone group was 0.054, which was very close to 0.05, suggesting that the correlation between BMI and mCS was stronger in the stand-alone group than that of the combined group. In addition, it seemed that there was no correlation between the extent of intervertebral space expansion (i.e., ΔDH) and mCS. Based on our data, we believe that the retraction force of the paravertebral ligament is almost negligible compared with the weight load.

The limitations of this study are as follows: (1) The sample size included in this study was still small, which may have caused bias. (2) The BMD T values were obtained before treatment, so the effect of antiosteoporosis treatment was ignored, which may have resulted in an underestimation of subsidence. (3) Due to the non-randomized grouping of the retrospective studies, significant differences in baseline BMD biased the comparison between the groups, but this did not interfere with the results of the multivariate linear regression analysis within each group.

## Conclusions

In conclusion, we suggest that patients with osteoporosis or osteopenia should avoid the stand-alone OLIF method, which may put them at greater risk of developing severe CS. OLIF combined with PSRF can significantly reduce FH loss and the risk of severe CS than the stand-alone OLIF method, which may benefit clinical outcomes in patients with osteoporosis or osteopenia.

## Data Availability

None.

## Abbreviations

PSRF: Pedicle-screw rod fixation
OLIF: Oblique lumbar interbody fusion
CS: Cage subsidence
BMI: Body mass index
BMD: Bone mineral density
VAS: Visual analog scale
ODI: Oswestry disability index
DH: Disk height
ΔDH: Disk height increment
FH: Foraminal height
tCS: Total cage subsidence
mCS: Main cage subsidence
